# Trustworthiness, Readability, and Suitability of Web-Based Information for Stroke Prevention and Self-Management for Korean Americans: Critical Evaluation

**DOI:** 10.2196/10440

**Published:** 2018-07-20

**Authors:** Mikyoung A Lee, Cha-Nam Shin, Kyungeh An

**Affiliations:** ^1^ College of Nursing Texas Woman's University Denton, TX United States; ^2^ College of Nursing and Health Innovation Arizona State University Phoenix, AZ United States; ^3^ Department of Adult Health and Nursing Systems School of Nursing Virginia Commonwealth University Richmond, VA United States

**Keywords:** stroke, website evaluation, trustworthiness, readability, suitability

## Abstract

**Background:**

Websites are common sources of health information to stroke survivors and caregivers for continual management of stroke and its long-term sequelae. The presence of risk factors and mortality rates related to stroke are high in Korean Americans. A vast majority of this group are active Web users and rely on the Web-based information due to lack of insurance and, thus, limited access to long-term stroke care. Thus, it is critical to evaluate existing stroke websites for their trustworthiness, readability, and suitability.

**Objective:**

The objective of our study was to provide a systematic evaluation of stroke-related websites regarding (1) trustworthiness, (2) readability, and (3) suitability for stroke prevention and self-management for Korean Americans.

**Methods:**

We selected a total of 156 websites using search terms “stroke,” “CVA,” “중풍 (jungpung),” and “뇌졸증 (noejoljung)” on Google and Yahoo. After eliminating duplicates and irrelevant websites (n=116), we evaluated a total of 42 websites (15 in English and 27 in Korean) using the National Library of Medicine’s health website’s evaluation tool for trustworthiness; Simple Measure of Gobbledygook for readability; and Suitability Assessment of Materials for suitability. All three instruments used the 3-point Likert scale: superior (=2), adequate (=1), or not suitable (=0).

**Results:**

Of the 42 websites evaluated, we rated 62% (26/42) websites as “adequate” or above for trustworthiness. The information on 48% (20/42) websites had not been updated for more than a year, which indicates poor currency; 33% (14/42) websites failed to provide the publisher and contact information, which yields poor authority; 50% (21/42) websites did not cite sources of health information, which indicates lack of accuracy. Only 2 websites met the recommended readability (5th grade or lower reading level). The suitability was also suboptimal; only 1 website was rated as “superior”; 60% (25/42) websites were “adequate,” and 38% (16/42) were “not suitable.” Most websites were limited in graphical directions, interactive motivations for desired healthy behaviors, and multiple language translations.

**Conclusions:**

The existing stroke-related websites in either English or Korean are trustworthy and suitable, yet precise citation of evidence-based information will improve trustworthiness. The contents requiring high reading level may set a barrier to the utilization of Web-based health information for Korean Americans with a lower level of education. In addition, supplementing graphical examples, interaction features, and culturally relevant information in multiple languages are the areas for improvement in suitability. The improved features can reduce the reading burden of stroke patients or caregivers and build their confidence when applying the information for stroke management in daily living. These strategies are especially crucial to Korean Americans, who inevitably seek Web-based information to fill the gap between their demand and access to health care for a long-term self-management after a stroke.

## Introduction

With advancements in the internet services and communication technologies, both release and seeking of health information through the Web have been exponentially growing. In the United States, 89% of adults use the internet, and among them, 72% lookup online for health information [1,2]. Use of mobile phones has made the access to health information ubiquitous. Recent surveys have reported that 77% of US adults own a mobile phone, and among them, 62% have used their phone to get information about a health condition [3,4].

Use of Web-based health information is common among Korean Americans [5-8], who constitute about 1.8 million and are the fifth largest Asian American population in the United States [9]. One possible reason for this population using websites as an important resource for health information may be the limited access to health care professionals due to lack of health insurance [10]. Korean Americans record one of the lowest rates of health insurance coverage among all racial and ethnic groups living in the United States [11-13]. The limited English proficiency, which is predominant in first-generation (75.5%) and monolingual (37%) immigrants, has also contributed to limited health care access among this group [14,15]. Limited health care access increases Korean Americans’ sense of self-responsibility and may lead individuals to be more active in seeking health information through available sources, such as Web-based information [10].

Universally, individuals with chronic or stigmatized diseases such as stroke are more likely to search for health information on the internet than those without the health conditions [16-19]. Stroke is the foremost cause of serious long-term disability, with high health care cost [20], and puts an increasing economic burden on health care resources [21]. The varying degrees of long-standing disability, as a result of stroke, lead to patients and their caregivers living with daunting long-term management. Stroke often results in emotional, cognitive, and physical impairments, which tend to be visible to others and cause stigmatizing social experiences after stroke [22,23]. With this burden, stroke survivors and caregivers often use websites as resources of information that they need [24].

Stroke is the third leading cause of death among Korean Americans [25]. The proportionate mortality ratios of stroke, especially hemorrhagic stroke, have been reported to be higher among Korean American women (2.07) and men (1.89) than among non-Hispanic white women (1.06) and men (0.94) [26]. Furthermore, the elderly have a higher prevalence of stroke risk factors, such as hypertension and dyslipidemia, than Caucasians, and the former lack knowledge about stroke [27]. Thus, prevention and appropriate long-term management of stroke and the sequelae with quality information is critical in this population.

Obtaining Web-based health information can be beneficial for or harmful to people. The explosion and proliferation of health information available online are on the promise that these online resources can confirm or broaden patients or families’ understanding of diseases and treatment opinions that influence health care decisions and empower them to effectively self-manage health conditions [28,29]. However, the quality of the Web-based health information, generally in trustworthiness, readability, and suitability aspects, is often questioned. Online health information seekers could be at risk of finding unreliable or inappropriate information from the websites. Incorrect or inappropriate health information can be used in an improper way and can cause detrimental outcomes by negatively influencing health care-related decisions [30,31]. As patients have a wide range of health information literacy, some patients are unable to critically assess health information or might misinterpret it [31]. Online health information can be advantageous only if it is understandable to the consumers [29].

Considering the high demand of Web-based health information in native or English language among Korean Americans, who have limited English proficiency, it is critical for the websites to be *trustworthy* with up-to-date, reliable, and accurate information, which is *readable* and *suitable* for this specific population. Thus, it is important to examine the trustworthiness, readability, and suitability of health information available in native and English languages on the websites. The study findings will facilitate devising strategies to better design and implement stroke-related websites.

The purpose of this study was to address the following question: Is the Web-based stroke information adequate for Korean Americans to read, understand, and engage in stroke care? Specifically, we aimed to evaluate stroke-related websites in terms of their (1) trustworthiness, (2) readability, and (3) suitability for stroke prevention and self-management in this group.

## Methods

### Selection of the Websites for Stroke

Initially, we considered a question that how Korean Americans would seek stroke information on the internet. One of the common behaviors exhibited was bilingual searches in their health information seeking as a technique for coping with limited English proficiency [32]. In general, people selected health websites within only the first one or two pages of search results (10 results per page) when searching the internet; about half of the internet users entered a single query [32,33]. In van Deursen and van Dijk’s [33] study, nobody used advanced search features (eg, Boolean operators like AND, OR, and NOT). In fact, Google and Yahoo have been the most popular search engines among Koreans and Americans [2,32].

Based on a review of these studies about online health information-seeking behaviors of Korean Americans, we searched stroke-related websites in Google and Yahoo search engines using the following terms: “Stroke,” “CVA,” “중 (jungpung),” and “뇌졸증 (noejoljung).” The term, “Stroke” is health care consumers’ preferred term listed in the Consumer Health Vocabulary Initiative [34]. The term, “CVA’” is another representative term for stroke. Furthermore, “중풍” and “뇌졸증” are the most commonly used Korean terms to indicate “stroke.” We retrieved a total of 156 stroke-related websites that appeared on the 1st and 2nd pages only after searching. With the use of terms “Stroke” and “CVA,” Google revealed 45 sites, while Yahoo led to 35 sites. Searching with the use of terms “중풍” and “뇌졸증” resulted in 36 sites from Google and 40 sites from Yahoo. Then, irrelevant websites, which were newsletters, commercial and noncommercial advertisements, and homonyms such as stroke motion in swimming or tennis, were excluded. After duplicates and the irrelevant sites (n=116) were eliminated, a total of 42 websites (15 in English and 27 in Korean), including those of the not-for-profit organizations, clinics, magazines, and blogs, were selected.

### Evaluation Tools

#### Trustworthiness

We initially evaluated each website for its trustworthiness using the following three criteria for health websites’ evaluation endorsed by the National Library of Medicine (NLM) [35]: currency or timeliness (when was the website last updated?); authority (who published the website?); and accuracy (are the sources cited reliable?). Each criterion was rated on a 3-point Likert scale, where 0=not suitable, 1=adequate, and 2=superior. The descriptions for each point are presented in Table 1. Thus, the possible total score per site ranged from 0 to 6, with higher scores indicating better trustworthiness.

#### Readability

We evaluated the readability of the information on selected websites using the Simple Measure of Gobbledygook (SMOG) Readability Test and the Reading Grade level of the Suitability Assessment of Materials (SAM) instrument. The SMOG has been validated as very easy to compute and provide a reasonably accurate measure of readability when evaluating consumer-oriented health care materials in many studies. We used the English version of the SMOG measure for evaluating the websites published in English [36] and the Korean version [37] for the websites in Korean. The formula requires counting 10 consecutive sentences at the beginning, middle, and end of website pages (30 total sentences). Second, it requires counting the number of words with ≥3 syllables in the 30-sentence sample. Then, with the total number of polysyllabic words counted, the grade level is determined using the SMOG conversion table. When using the SAM, the reading grade level can be measured by using different reading formulas, including the SMOG. Studies on the readability of patient education materials have often used both the SMOG and SAM tools together. For example, Rosenfeld et al [38] and Shieh and Hosei [39] computed the SMOG scores and integrated the scores to SAM 3-point categories. Similarly, in this study, the grade levels computed by the SMOG formula were converted to the SAM’s 3-point scale, where 0=not suitable (9th-grade level and above), 1=adequate (6th-, 7th-, or 8th-grade level), and 2=superior (5th-grade level or lower).

#### Suitability

Each website was evaluated by the SAM instrument [40]. The SAM has been used in many studies to evaluate the suitability of online health information [41-46]. It consists of 22 items grouped under 6 factors, namely (1) Content, (2) Literacy Demands, (3) Graphics, (4) Layout and Typography, (5) Learning Stimulation and Motivation, and (6) Cultural Appropriateness. We added one item to the list, which was “multiple language translations,’’ under the factor of Cultural Appropriateness. Each of the 23 items was rated on an ordinal scale, where 0=not suitable, 1=adequate, and 2=superior. Raw scores were summed to yield an overall score. This overall score was then converted to a percent of the possible total score for each website using the following formula: converted percent=total score/total possible score (46=23 items×2 maximum score per item)×100. A converted percent of 70%-100% indicates a superior website, 40%-69% indicates an adequate website, and 0%-39% indicates a not suitable website [40].

When previous studies assessed the suitability of health information across multiple sources, the converted percent was also applied to the present the quality of the health information per SAM criterion [47,48]. In this study examining which criterion was suitably met across the websites, the mean score per criterion was computed and then converted to a percent of the possible best score using the following formula: the converted percent=([n of websites×0 point]+[n of websites×1 point]+[n of websites×2 points])/Total possible best score (84=42 websites×2 points)×100.

Then, if the converted percent for a criterion was in the range of 70%-100%, the quality of the information regarding the criterion was considered as “superior” across websites. If the percentage was in the range of 40%-69%, the quality of the information per criterion was considered at an “adequate” level. If the percentage was in the range of 0%-39%, the information regarding a criterion was considered as “not suitable” across the websites.

### Evaluation and Analyses

Three content experts evaluated each website independently in the first round and collected the ratings. In the second round, the three raters reviewed the websites synchronously to ensure the accuracy of understanding of each criterion and, then, shared their rationales for the ratings. The frequencies and intraclass correlation coefficients (ICCs) per the evaluation criteria were obtained using SPSS 24. The final interrater agreement levels for the three evaluation criteria were as follows: NLM, ICC=.969-.987; SMOG, ICC=.810; and SAM, ICC=.626-.994.

## Results

### General Characteristics

Out of the 42 appraised websites, 15 stroke websites were in English and 27 were in Korean. Of all, 12 websites were those of the not-for-profit organizations, 7 were blogs on stroke, 5 were published by clinics or hospitals, 5 were Wikipedia pages, 4 were medical magazine sites, 3 were general magazine sites, 2 were postings on broadcasting websites, 1 was a pharmacological company website, 1 was an insurance company website; 1 was a general online forum site, and 1 was a medical information website.

### Trustworthiness

Overall, 62% (26/42) websites received a rating of >3.0, which presents “adequate” level. Regarding the currency or timeline, 79% (33/42) of the selected websites indicated published date of the information on their websites, whereas 21% (9/42) did not indicate a published date of the information. The updated dates of the information on 31% (13/42) websites were <1 year old, while on 48% (20/42) websites these were older than 1 year. In terms of authority, 21% (9/42) websites did not present the publisher or contact information. Regarding accuracy, 33% (14/42) websites posted health information based on medical research evidence along with citations, whereas 50% (21/42) of the websites did not cite any source of information (Table 1).

### Readability

A total of 19 of 42 (45%) websites were presented at the 9th-grade level or above (0=not suitable). Twenty-one (50%) websites were presented at reading levels between 6th and 8th grade (1=adequate). Only 2 websites (5%) were presented at the 5th grade- or lower level (2=superior).

### Suitability

Table 2 presents the frequencies per rating score, ICC of the three raters per the SAM criterion, and the converted suitability percent score. The overall converted suitability percent score of all 42 websites was 55.7%, which represents “adequate” suitability. Out of 42 websites, only 1 website of the American Stroke Association was rated as “superior”; 60% (25/42) websites were “adequate,” and 38% (16/42) websites were “not suitable.” The websites were superior in the quality of layout and typography (85.3%). Furthermore, the websites were at an adequate level regarding the Content (65.5%); Literacy Demands (68.6%); Graphic Illustration, Lists, Tables, and Charts (42.4%); and Learning Stimulation and Motivation (47.2%), even though there were some individual items with inadequate suitability. However, Cultural Appropriateness was not suitable (22.2%).

Under the Content category, the quality of purpose and scope was superior, with scores of 90.5% and 79.8%, respectively. Regarding the extent of the content topics, 26% (11/42) websites aimed at desirable behavior rather than at nonbehavioral facts. In addition, 60% (25/42) websites showed <40% of the content topics focusing on desirable behaviors or actions. However, 6 websites did not present such contents. Many of the appraised websites (23/42, 55%) did not present summaries or reviews well to convey key messages.

Regarding the Literacy Demands, the selected websites were suitable in writing style (81.0%), sentence construction (77.4%), vocabulary use (72.6%), and organization using road signs (82.1%). However, the reading grade level was not suitable across websites (29.8%).

**Table 1:** Trustworthiness of stroke websites by the National Library of Medicine criteria.

Evaluation criteria^a^			Frequency, n (%)	ICC^b^
**Currency or timeliness: when was the website last updated?**				.969
	Superior (2)	The published date of the information is indicated and is less than 1 year old (last year)	13 (31)	
	Adequate (1)	The published date of information is indicated, but is older than 1 year	20 (48)	
	Not suitable (0)	No indication	9 (21)	
**Authority: who published the website?**				.980
	Superior (2)	The publisher’s information (individuals or organizations) and contact information can be easily found	28 (67)	
	Adequate (1)	The publisher’s information (individuals or organizations) can be found. But there is no contact information	5 (12)	
	Not suitable (0)	No indication	9 (21)	
**Accuracy: are the sources cited reliable?**				.987
	Superior (2)	The information is drawn based on sound medical research, and the information sources are cited	14 (33)	
	Adequate (1)	The information sources are cited but not based on medical research	7 (17)	
	Not suitable (0)	No indication	21 (50)	

^a^Score shown in parentheses.

^b^ICC: intraclass correlation coefficient.

**Table 2:** The evaluation of the stroke websites based on Suitability Assessment of Materials.

Category and criteria		Frequency, n (%)			ICC^a^	Converted %^b^
		Not suitable (0)	Adequate (1)	Superior (2)		
**Content**						
	Purpose	0 (0)	8 (19)	34 (81)	.626	90.5
	Scope	2 (5)	13 (31)	27 (64)	.896	79.8
	Content topics	6 (14)	25 (60)	11 (26)	.875	56.0
	Summary and review	23 (55)	8 (19)	11 (26)	.993	35.7^c^
	Average					65.5
**Literacy demands**						
	Reading grade level	19 (45)	21 (50)	2 (5)	.810	29.8^c^
	Writing Style	1 (2)	14 (33)	27 (64)	.859	81.0
	Sentence construction	1 (2)	17 (41)	24 (57)	.727	77.4
	Vocabulary	2 (5)	19 (45)	21 (50)	.746	72.6
	Learning enhancement by advance organizers	6 (14)	3 (7)	33 (79)	.962	82.1
	Average					68.6
**Graphic illustration, lists, tables, and charts**						
	Cover graphic	20 (48)	13 (31)	9 (21)	.950	36.9^c^
	Type of illustrations	14 (33)	15 (36)	13 (31)	.950	48.8
	Relevance of illustrations	15 (36)	11 (26)	16 (38)	.975	51.2
	Graphical direction: lists, tables, charts, and forms	25 (60)	10 (24)	7 (17)	.956	28.6^c^
	Captions are used to announce or explain graphics	14 (33)	17 (41)	11 (26)	.976	46.4
	Average					42.4
**Layout and typography**						
	Typography	1 (2)	9 (21)	32 (76)	.863	86.9
	Layout	1 (2)	12 (29)	29 (69)	.813	83.3
	Subheadings and chunking	4 (10)	4 (10)	34 (81)	.965	85.7
	Average					85.3
**Learning, stimulation, and motivation**						
	Interaction included in the text or graphics	16 (38)	15 (36)	11 (26)	.967	44.0
	Desired behavior patterns are modeled	14 (33)	12 (29)	16 (38)	.975	52.4
	Motivation	16 (38)	14 (33)	12 (29)	.965	45.2
	Average					47.2
**Cultural appropriateness**						
	Cultural match: logic, language, experience	24 (57)	8 (19)	10 (24)	.992	33.3^c^
	Cultural image and examples	29 (69)	10 (24)	3 (7)	.987	19.0^c^
	Multiple languages translation	36 (86)	0 (0)	6 (14)	.994	14.3^c^
	Average					22.2^c^
Overall average						55.7

^a^ICC: intraclass correlation coefficient.

^b^Converted Percent=([n of websites×0 point]+[n of websites×1 point]+[n of websites×2 points])/total possible best score (84=42 websites×2 points)×100. Interpretation: 70%-100%=superior; 40%-69%=adequate; 0%-39% =not suitable.

^c^Information rated as “not suitable” per criterion across the evaluated websites.

The overall suitability of the Graphic Illustration, Lists, Tables, and Charts category was rated as “adequate” (42.4%). In this category, the type of illustrations (48.8%), relevance of illustrations (51.2%), and captions (46.4%) were “adequate” at the lower levels, but cover graphic (36.9%) and graphical direction (28.6%) were “not suitable.” In more detail, 48% (20/42) of the websites did not present cover graphic to attract attention and to clearly portray the purpose of the website.

In addition, 60% (25/42) of the websites presented graphics without explanation and 24% (10/42) showed too brief “how to” directions with graphics for readers; only 17% (7/42) websites provided step-by-step directions with an example that will build self-efficacy (confidence). The layouts and typography were superiorly presented in most appraised websites, with the converted suitability scores of 83.3%-86.9%.

In the Learning Stimulation and Motivation category, the suitability of each criterion was rated as “adequate,” even though the suitability percent scores were not high, ranging 44.0%-52.4%. In more details, 38% (16/42) websites did not provide interaction learning or stimulation and 36% (15/42) used passive interactions with the Questions & Answer format. Regarding the desired behavior pattern presentation, 38% (16/42) websites demonstrated instructions for specific behavior and skills by using specific, familiar instances with the rating of “superior.” In addition, 29% (12/42) websites were rated as “adequate” as they provided the information in a mix of technical and common language that the reader may not easily link with daily living activities. In terms of Motivation, only 29% (12/42) websites reached the “superior” level, which means that complex topics are subdivided so that readers may experience small successes in understanding or problem solving, leading to self-efficacy (confidence). Furthermore, 38% (16/42) websites did not have features of motivation.

The Cultural Appropriateness across websites was “not suitable”; the converted suitability percent scores ranged from 14.3% to 33.3%. Regarding cultural match, 57% (24/42) websites did not present the information in a culturally similar logic, language, and experience of the target population culture. The cultural image and examples were rarely shown in 69% (29/42) websites. Moreover, 86% (36/42) of the websites were presented in only one language and could not be translated into other languages.

## Discussion

### Principal Findings

This study is the first to provide a systematic evaluation of the stroke-related websites considering Korean Americans. The findings of this study present valuable information about the trustworthiness, readability, and suitability of stroke-related English and Korean websites, which were trustworthy and marginally suitable, but not easily readable. In addition, areas that need improvement were identified in each criterion. This information can be utilized by clinicians and researchers in improving or designing stroke websites as well as by people who seek stroke information in their self-assessment of the quality of the information on websites.

### Trustworthiness

Patients and caregivers look up the websites as they are easy to access, not expensive, and, in general, believed to be timely [32]. Patients are often unsure about which websites to trust and are concerned about accessing potentially misleading or illegitimate health information [49,50]. The outdated, inaccurate, and unreliable Web-based information can mislead the public in their understanding of stroke and stroke management. In the limited chance of education on which website and its information are trustworthy, Korean Americans consider merely the repetition of specific information as a criterion for evaluating the trustworthiness of websites; if the same information appears in several locations on the internet, Korean Americans are likely to simply believe that the information or the website is reliable [32]. The trustworthiness of the website information could be easily checked by the NLM’s [35] three criteria of health website evaluations. These evaluation criteria need to be educated to online health information seekers, including the population in question.

In this study, the information on 20 websites was published more than 1 year ago and 9 websites did not indicate a published date of the information. Notably, half of the websites did not cite any source of information on their websites. Agarwal et al [30] also found in their evaluation of educational resources in 3 stroke-related websites that users criticized the lack of citations and references for the material and facts provided on all 3 websites. It is important to present the valid and reliable primary information with the published date and the information sources for health consumers to help them with right decision making regarding their health issues [16,30]. It is recommended to scrutinize or peer review before presenting the information on the website. Health care providers should be aware of, at least, these three criteria when providing health care information to patients and caregivers via papers or websites. Furthermore, health care providers should provide opportunities to educate patients and caregivers on how to check and whether to rely on health care information in any type of Web information platform.

### Readability

Reading level is an essential component of health literacy and is included as one of the Literacy Demand criteria in the SAM tool. Concerned about health literacy, the Joint Commission [51] has recommended patient education materials to be written at or below the 5th-grade level and has developed an action plan to promote patient literacy. In this study, 19 out of 42 websites were written at the 9th-grade level or above, which is rated as “not suitable” and categorized as “difficult” in the US readability categories [52]. Only 2 websites were written at the 5th grade- or lower level. This result is consistent with those of previous studies, which evaluated some stroke-related websites: the mean readability level of stroke information was found to be 10th grade by Griffin et al [53] in their evaluation of 30 stroke educational websites; these websites were different from the set of websites appraised in our study, except 3 overlapping websites. Furthermore, Sharma et al [24] found that over half of 100 stroke websites were produced at the 12th-grade level or above when the readability was measured with the SMOG. The high reading levels may be due to complex medical terminologies without explanations or translation into layperson’s terms.

The health information is only useful if the consumer can comprehend the presented information. The consequences of not understanding health information can negatively affect both a person’s health and his or her utilization of health care services. The use of consumer health vocabulary may help lower the reading grade level on both English and Korean stroke websites. In the United States, the Open-Access, Collaborative Consumer Health Vocabulary provides 156,826 consumer-friendly health phrases and synonyms for professional clinical terms [34,54]. In South Korea, a consumer vocabulary system for health information was developed in a study [55]. Also, the complex medical terms or sentences could be provided with additional understandable explanations so that readers become familiar with them and can use them appropriately [38]. Korean Americans’ limited English proficiency should also be taken into consideration. Limited English proficiency was identified as a critical source of health vulnerability; it impacted health perceptions, led to higher health risk, and negatively influenced health care utilization [56-58]. Those with limited English proficiency scored lower on all measures of health conditions than their English-proficient counterparts [57]. Considering the limited English proficiency among Korean Americans, terms and sentence structures of stroke care information on English websites should be used at an appropriate reading level.

### Suitability

The overall suitability of all 42 websites was rated as “adequate” (55.7%); however, there are needs for improvement in many areas. Regarding the Content, the majority of the websites explicitly showed the purpose and the scope of the content. However, they did not present summaries or reviews well to convey key messages. In order to ensure the delivery of important messages and to assist the readers’ comprehension, it is necessary to retell key messages in different words or examples and provide their summaries.

The overall suitability of Graphic Illustration was marginally adequate (42.4%). Half of the websites did not present cover graphic to attract attention and clearly portray the purpose of the websites. In addition, 60% (25/42) of the websites presented graphics without explanation and 24% (10/42) showed too brief “how to” directions for readers. This finding is similar to the lack of graphics and other nontextual media covering patient education materials in Agarwal et al’s [30] evaluation of educational resources in 3 stroke-related websites. Images and videos combined with the text can act as supplements to difficult topics covered on the site [30] and can increase the ability of the user to understand and retain the material [59]. Since the medical information is relatively unfamiliar to laypersons, and the reading level is high, step-by-step directions with graphical examples can reduce the reading burden of stroke patients and caregivers. Furthermore, use of strategies to enhance readers’ understanding of the information on websites is especially important to Korean Americans, who inevitably seek online information due to lack of insurance and limited English proficiency.

The Learning Stimulation and Motivation category also received the rating of “adequate,” but it was not high (47.2%). No interaction learning or stimulation was provided on 38% (16/42) websites. Regarding the desired behavior patterns presentation, the criterion of the “superior” rating probed if websites demonstrated instructions for specific behavior and skills by using specific, familiar instances; more than half of the websites failed to reach this level. Only 29% (12/42) websites ranked “superior” in the Motivation criterion. Mere access to Web-based health information does not necessarily empower consumers and patients. Providing actionable health information with accuracy is the top vision in the National Action Plan to Improve Health Literacy published by the US Department of Health and Human Services [60]. In addition, practical guidelines demonstrating specific behavior and skills applicable to daily living will motivate the readers to experience small success in understanding or problem solving, leading to self-efficacy. Multimedia and interactivity features can assist information users to build more confidence by tailoring to individual needs and progress of condition management and rehabilitation. The incorporation of self-assessments of risks or symptom changes and modeling of desirable health behaviors into the website will promote self-efficacy in learning. Miller and Leroy [54] suggested that it would be ideal if a consumer would go to a website, answer a few questions, and in a few seconds, a document written specifically for their needs and appropriate to their reading skill would appear in their Web browser. The features of dynamic discussions and communication will lead to co-learning with each other [28,61]. Chat rooms and email services with health care providers on websites are other recommendable features to satisfy patients’ information needs.

Of all, 86% (36/42) websites were presented in only one language and could not be translated into other languages. More than half of the websites in English or Korean did not present information matched to the Korean culture in texts, images, or examples. The cultural factors influence health information acquisition and access to social support for ethnic minorities [62]. Korean consumers rely heavily on Korean-specific health information, regardless of education levels [32]. Korean Americans have a lower level of knowledge on stroke risk factors and symptoms than the general US population [63]. Websites are important sources and channels of health information for them. If websites provide more diverse and culturally appropriate information in both Korean and English, the health information can be delivered to a broader population of Korean Americans. Then, it may contribute to increase in knowledge about stroke prevention and self-management among this group.

### Limitations

We evaluated the websites that appeared on the first and second pages only on Google and Yahoo browsers. Thus, the results cannot be generalized to reflect the features of all stroke-related websites. However, these websites can be the most popular or important ones because studies have found that 73%-95% of Web users never view past the first search engine results page and have emphasized the value of the first page [64-67]. In addition, it may indicate the increasing ability of Web search engines to retrieve the relevant Web-based information more effectively [64]. The websites evaluated in this study were stroke specific, which may limit the application of this study results to websites of other specific health conditions.

### Conclusions

The quality of health information for patients with stroke is vital to ensure the good recovery and to improve the quality of life. Easily obtainable, user-friendly, accurate, and reliable online resources could help people make appropriate decisions about how best to maintain or improve their health condition.

We found that the existing stroke-related websites in either English or Korean are trustworthy and suitable. However, it was notable that the accuracy of information on these websites was low due to lack of citations and references. Providing accurate, reliable, high-quality, and evidence-based information is a critical responsibility of health professionals. In addition, the study findings signified the need for diverse features to reduce the high reading level of the information on the websites. Graphical examples, multimedia, and interactive features can reduce the reading burden of stroke patients and caregivers, as well as build more confidence or self-efficacy when applying the information for condition management and rehabilitation in daily living. Rather than posting the information in a nonspecific way, practical guidelines demonstrating specific behavior and skills applicable to daily living will motivate the reader to experience small success in stroke self-management, leading to self-efficacy.

These strategies to enhance readers’ understanding of Web-based information are especially important to Korean Americans, who inevitably seek online information due to lack of familiarity with the US health care system, inadequate health insurance coverage, and language barriers. Culturally sensitive, high-quality health information relevant to this group through websites may have great potential to increase the knowledge of stroke self-management and make significant contributions to promote the health of Korean American stroke survivors. In the future, the evaluation of stroke websites by Korean American laypersons using the evaluation tools used in this study might be an educational health literacy intervention to learn how to evaluate and consider the design and content aspects in the websites. The examination of how this population navigates stroke websites and apply the health information obtained from these websites for their daily health behaviors should be considered for future studies.

## Abbreviations

CVA: cardiovascular accident
ICC: intraclass correlation coefficient
NLM: National Library of Medicine
SAM: Suitability Assessment of Materials
SMOG: Simple Measure of Gobbledygook
